# Qigong Exercise May Reduce Serum TNF-α Levels and Improve Sleep in People with Parkinson’s Disease: A Pilot Study

**DOI:** 10.3390/medicines4020023

**Published:** 2017-04-23

**Authors:** Sanghee Moon, Marshall Schmidt, Irina V. Smirnova, Yvonne Colgrove, Wen Liu

**Affiliations:** 1 Department of Physical Therapy and Rehabilitation Science, University of Kansas Medical Center, Mailstop 2002, 3901 Rainbow Blvd., Kansas City, KS 66160, USA; ismirnova@kumc.edu (I.V.S.); ycolgrove@kumc.edu (Y.C.); 2 Bioengineering, University of Kansas, 1530 W 15th St., Lawrence, KS 66045, USA; m333s074@ku.edu

**Keywords:** mind–body exercise, Qigong, six-healing sounds, Parkinson’s disease, sleep, inflammatory cytokines, TNF-α

## Abstract

**Background:** Inflammatory cytokine levels are often elevated in people with Parkinson’s disease (PD). People with PD often experience sleep disturbances that significantly impact quality of life. Past studies suggest inflammatory cytokines may be associated with various symptoms of PD. Benefits of Qigong, a mind–body exercise, have been shown in different neurological conditions, but there is still a lack of clinical evidence in the PD population. **Methods:** Ten people with PD were recruited and randomly assigned into two groups receiving six weeks of Qigong (experimental group) or sham Qigong (control group) intervention. The levels of TNF-α, IL-1β, and IL-6 in subjects’ serum and sleep quality were measured before and after the intervention. **Results:** After the intervention, the serum level of TNF-α in the experimental group was significantly decreased in all subjects, while the level in the control group showed a trend to increase. Qigong exercise significantly improved sleep quality at night. There was a strong correlation between changes in the level of TNF-α and sleep quality. **Conclusion:** Qigong exercise decreased TNF-α level in people with PD and helped improve sleep quality. TNF-α may have a potential to influence the sleep quality in people with PD.

## 1. Introduction

Parkinson’s disease (PD) affects over one million people in the United States, causing a considerable burden on patients and the society [1,2]. PD is a neurodegenerative disorder characterized by a disruption of dopaminergic neurons and formation of Lewy bodies in the basal ganglia [3]. Although the underlying physiological mechanisms have not been elucidated, inflammatory cytokines are closely associated with PD pathogenesis. A study examining postmortem PD brains found that the levels of various pro-inflammatory cytokines including interleukin (IL)-1β, IL-6, tumor necrosis factor (TNF)-α, and others were significantly elevated in the striatum and in lumbar cerebrospinal fluid (CSF) [4]. Another study demonstrated that the levels of pro-inflammatory cytokines including IL-1β, IL-2, IL-4, IL-6, and TNF-α were elevated in CSF in people with PD [5].

Sleep dysfunction, one of the more common non-motor symptoms in PD, significantly impacts quality of life in PD [6,7,8]. Up to 96 percent of people with PD experience at least one sleep-related dysfunction [9]. Compared to elderly people who do not have PD, people with PD have been reported having more frequent sleep dysfunctions including daytime sleepiness, insomnia, night-time waking, restless leg syndrome, vivid and/or violent dreams, sleep apnea, and periodic limb movements of sleep [7,8,10,11,12,13].

IL-1β and TNF-α are involved in sleep-wake regulation [14], and IL-6 and TNF-α may contribute to sleepiness and fatigue [15]. Increased IL-6 and TNF-α levels may promote depression by downregulating serotonin metabolism [16]. However, no study so far has examined the relationship between sleep and inflammatory cytokines in people with PD.

Qigong exercise is a mind–body intervention rooted in classic Chinese medicine, which has been practiced for thousands years to improve health and prevent illnesses [17]. Benefits of Qigong exercise in neurological disorders have been reported in past studies from our and other research groups. Qigong exercise improved motor and non-motor symptoms in people with PD [18,19]. Qigong exercise has beneficial effects on immune system in healthy adults [20]. However, there have been no studies that have examined the effect of Qigong exercise on inflammatory status in people with PD.

The present pilot study was designed to evaluate changes in the serum levels of inflammatory biomarkers pre- and post-Qigong exercise intervention. Additionally, possible associations were examined between changes in sleep quality measurements and changes in inflammatory biomarkers in people with PD pre- and post-Qigong exercise intervention.

## 2. Materials and Methods

### 2.1. Subjects

A total of ten subjects with mild to moderate PD (Hoehn and Yahr (HY) stage ranging from 1 to 3) were enrolled. All enrolled individuals were screened and included in the study if they met the following criteria: diagnosis of idiopathic PD, between 40 and 75 years of age, no deep brain stimulation surgery, and no anticipated changes to PD medication within the study period. We excluded people with mini mental state examination score <24, neurological diseases other than PD including other forms of PD, uncontrolled or significant cardiovascular diseases, deep brain stimulation, and expected change in PD medications over the course of the study.

Subjects were assigned into either a control (*n* = 5) or an experimental (*n* = 5) group by way of random allocation using computer-generated random numbers. The subjects and assessors were blinded to random assignment. Subjects in the experimental group learned and performed a Qigong exercise, while subjects in the control group learned and performed a sham Qigong exercise. Prior to participation in the study, every subject signed an informed consent approved by the Institutional Review Board of the University of Kansas Medical Center (KUMC).

After the informed consent was signed, basic medical history information was collected and the baseline assessment that included blood sampling and collection of clinical outcomes was performed for each subject. There were two introductory training sessions in the following two weeks where subjects learned the exercises. During the following six-week intervention period, subjects in each group met once per week to have a group exercise session, and were asked to practice the learned exercise twice daily at home. Post-intervention testing, which consisted of the same evaluations as baseline assessments, was conducted within two weeks after the six-week intervention period ended.

### 2.2. Intervention

The subjects in the experimental group were taught the “six-healing sounds” Qigong exercise by an experienced trainer (Table 1). The Qigong exercise can be performed while standing, sitting, or lying down. If practiced properly, one session takes between 15 and 20 min. After two introductory training sessions, the subjects in the intervention group were instructed to perform the exercise twice daily at home, once in the morning upon waking up and a second time at night just before going to bed. In addition to home-based Qigong exercise, subjects in each group met once weekly for a group exercise session. This 45- to 60-min session provided an opportunity for the instructor to observe and correct performance of the subjects, answer questions, and encourage group discussion of relevant issues. To monitor compliance, subjects were asked to maintain exercise diaries that were reviewed at weekly group sessions.

The control group followed a similar set of instructions, but learned a sham Qigong exercise instead. The physical movements of the sham Qigong exercise were the same as in real Qigong exercise. The sham Qigong exercise, however, did not include the deep breathing technique or meditation, nor did the subjects utter the sounds associated with the movements. These subjects likewise kept a weekly exercise diary throughout the experimental period and turned them in during group sessions. The weekly sessions consisted of group exercise and discussion of relevant issues.

### 2.3. Serum Sampling

Blood samples from all subjects were taken before and after the Qigong intervention. About 20 mL of venous blood samples were collected at each time using BD Vacutainer^®^ serum blood collection tubes (Becton Dickinson; Franklin Lakes, NJ, USA) by a registered nurse. Immediately after blood drawing, blood samples were stored at room temperature for 15 to 20 min and centrifuged at 500× *g* for 5 min to separate serum from blood cells. Only serum, the top part of centrifuged blood samples, was aspirated and transferred into clean 10 mL test tubes, stored at −80 °C, and later aliquoted for storage at −80 °C until assaying.

### 2.4. Biomarker Assays

Three commercially available enzyme linked immunosorbent assay (ELISA) kits (Thermo Fisher Scientific, Inc.; Waltham, MA, USA) were used to detect the levels of human cytokines that included IL-1β (catalog number EH2IL1B), IL-6 (catalog number EH2IL6), and TNF-α (catalog number EH3TNFA). The assay ranges for IL-1β and IL-6 ELISA kits were from 10.2 to 400 pg/mL and for TNF-α was from 15.6 to 1000 pg/mL. All the assays used in this study were performed accordingly to the protocols provided by the manufacturer.

### 2.5. Sleep Quality Measurements

To measure the quality of sleep, the Parkinson’s Disease Sleep Scale 2 (PDSS-2) was administered [8,21]. This scale consists of a total of 15 items that assesses motor problems at night, PD symptoms at night, and disturbed sleep [21]. The validity and reliability of PDSS-2 has been proven by various studies [22,23,24].

### 2.6. Statistical Analysis

Paired and unpaired *t*-tests were used to compare the experimental and control groups in the levels of measured inflammatory biomarkers and PDSS-2 sub-scale and overall scores before and after the intervention. Pearson’s correlation coefficients were calculated to find a possible correlation between the levels of inflammatory biomarkers and the PDSS scores.

## 3. Results

### 3.1. Subject’s Characteristics

Eight out of ten subjects completed all sessions of the study. One subject in each group dropped out. At baseline, there were no significant differences between the experimental and control groups in all measures including age, disease duration, symptom duration, and HY stage (Table 2). Compliance to home exercise sessions were 80.5 ± 31.9% for the experimental group and 91.7 ± 3.4% for the control group, which were not significantly different.

### 3.2. Effects of Qigong Exercise on Inflammatory Status and Sleep Quality in PD

At baseline, the levels of TNF-α were not significantly different in the experimental group and in the control group (Table 3). After the intervention, the level of TNF-α in the experimental group was significantly reduced (*p = 0.036*), while the level of TNF-α in the control group showed a trend to increase. Regarding IL-1β and IL-6 levels, despite using ELISA kits allowing for detection of as little as 10.2 pg/mL of the cytokine, we were unable to detect IL-1β or IL-6 either pre- or post-intervention.

The results of the PDSS-2 scores are shown in Table 4. Between the experimental and control groups, no significant differences were found at baseline evaluation. Mean changes (Δ = post-score – baseline-score) in the PDSS-2 total and sub-scale scores in the experimental group showed a negative trend, while the control group showed a positive trend. Significant differences were found in the changes in PD symptoms at night (*p = 0.036*) and total PDSS-2 score (*p = 0.0003*).

### 3.3. Relationships Between TNF-α Levels and Sleep Disturbances in PD

**Figure 1:**
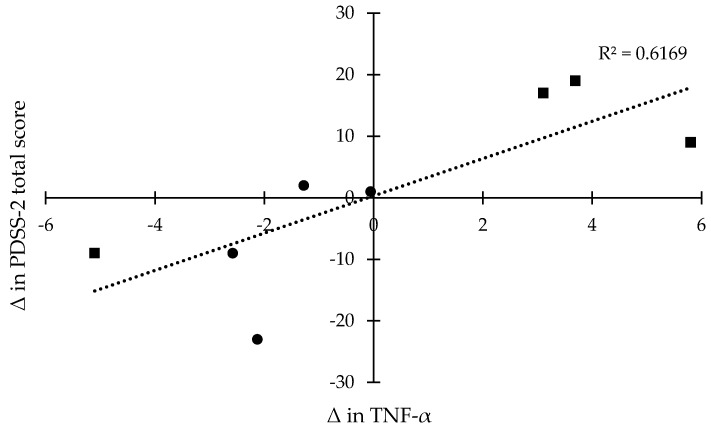
Correlation coefficients (r) between mean changes (Δ; Δ = post-score – baseline-score) in the levels of TNF-α and mean changes (Δ) in PDSS-2 scores in the experimental (●) and control groups (■).

Pearson’s correlation coefficients (*r*) were calculated between the mean changes (Δ) in the PDSS-2 scores and in the levels of TNF-α. Overall, there was a significant correlation between the mean changes (Δ) in TNF-α level and in total score of PDSS-2 with a correlation coefficient of 0.7854 (Figure 1). For the mean changes (Δ) in the sub-scale scores of PDSS-2 related to the mean changes (Δ) in TNF-α level, the correlation coefficients were 0.6133 for motor symptoms at night, 0.6546 for PD symptoms at night, and 0.6687 for disturbed sleep.

## 4. Discussion

The results of this pilot study indicate that Qigong exercise may reduce serum level of TNF-α in people with PD. This new finding may be clinically important. There are several key observations in this study. First, TNF-α is found commonly elevated in PD studies [25,26,27]. Although there were no healthy subjects for comparison, the elevated levels of TNF-α in our subjects were indicated through a comparison to data reported in the literature. The baseline mean value of TNF-α level in our subjects was 13.07 pg/mL, which is much higher than what has been reported in the healthy population, such as 0 pg/mL in 24 healthy subjects [28], 8.7 pg/mL in 140 healthy subjects [29], and 2.5 pg/mL in 23 healthy subjects [30]. Second, inflammatory cytokines, including IL-1β, IL-6, and TNF-α, are known to play an important role in PD symptoms and progression. Microglial cells mediate the immune reaction in the central nervous system and can be quickly activated by environmental stimuli. Activated glial cells express pro-inflammatory cytokines such as IL-1β, IL-6, and TNF-α. In PD, overexpression of inflammatory cytokines results in a decrease of dopaminergic cells in the brain, which leads to PD symptoms and disease progression [31]. An increase in the levels of inflammatory cytokines is often associated with neurodegeneration as shown in people with PD and Alzheimer’s disease [32]. Increased IL-6 and TNF-α levels may also promote depression by downregulating serotonin metabolism [16]. Finally, any decrease in the levels of inflammatory cytokines such as IL-1β or TNF-α may help reduce some PD symptoms, since inflammatory cytokines play an important role in sleep regulation and fatigue [14,15]. In fact, non-motor symptoms such as cognitive function, depression, and anxiety were correlated with levels of inflammatory cytokines in serum in people with PD [33,34].

Our results indicate also that Qigong exercise may improve sleep quality in people with PD. Sleep dysfunction in the form of motor symptoms at night, PD symptoms at night, and disturbed sleep, as evaluated by PDSS-2, are common to people with PD [6,7,8,21]. In our previous study in a single group of people with PD, we observed a significant improvement in sleep quality after Qigong exercise as compared to baseline measurement [18]. The current pilot study showed significant improvement in sleep quality in people with PD after Qigong exercise as compared to the control group. Qigong and Tai Chi exercises, both of which are considered as forms of meditative movement [35], have shown significant benefits in the sleep quality in people with fibromyalgia [36,37], chronic fatigue syndrome [38], moderate sleep disturbance and daytime sleepiness [39], and cardiovascular disease [40]. Meditation, one of the key components of Qigong exercise, may also play a role in the improvement in sleep quality in the experimental group. In our study, the experimental group practiced meditation with abdominal breathing technique, while the control group did only body movements. In the previous studies, mindfulness meditation improved sleep quality in older adults with sleep disturbances [41] and cancer patients [42,43].

The results of the current study have shown a strong correlation between changes in TNF-α concentration in serum and changes in the PDSS-2 total score. Menza and colleagues (2010) suggested that TNF-α may play a role in the development and maintenance of non-motor symptoms in PD, since TNF-α was consistently associated with sleep and other non-motor symptoms [44]. Our findings may imply a possible contribution of reduced inflammatory cytokine levels to the improvement in sleep quality in people with PD. Changes in TNF-α levels also were observed after aerobic exercise with moderate-intensity in people with PD [45], and in breast cancer survivors after participating in a yoga program [46]. However, there has been no systematic research correlating changes in inflammatory cytokine levels and symptoms in people with PD after an intervention. Future research is required to explore the issue.

The current study has several limitations. First of all, the small sample size in this pilot study is a significant limitation. Furthermore, we were unable to detect IL-1β and IL-6 using specific ELISA kits. This may be due to unintended human error while preparing test samples or conducting the assays. It is likely, however, that the concentrations of IL-1β and IL-6 in our samples were below the lowest detection limit (10.2 pg/mL) of the ELISA kits employed. Some other studies were also completely or partially unable to detect the concentrations of IL-6 [28,34]. In addition, we did not control for subject’s activity level prior to blood draws. Concentration of IL-6 in plasma can dramatically change during and after physical activity or exercise [47]. We may need to control subject activity level to avoid any upsurge of IL-6 concentration after physical activities. Finally, there is no consensus on the length of Qigong exercise to improve a particular condition. Future studies with longer intervention periods are warranted to determine whether longer practice would further improve sleep or other symptoms.

## 5. Conclusions

Qigong exercise may reduce levels of TNF-α in serum and improve sleep quality for people with PD. The improvement of sleep quality through Qigong exercise may be associated with changes in the inflammatory status and the immune system in general. However, this study was a pilot feasibility study with a limited number of subjects to examine the feasibility of Qigong exercise for people with PD, especially with regard to improving sleep quality and exploring the changes in inflammatory biomarkers that are known to be associated with PD. Future studies with a larger sample size are needed to confirm and extend findings of the current study. A wider selection of studied inflammatory biomarkers will be beneficial to reveal a clearer association between the inflammatory status and improvement in sleep quality induced by Qigong exercise.

## Figures and Tables

**Table 1:** Description of six-healing sounds Qigong exercise.

Movement	Sound	Body Movement and Breathing
Adjusting movement	No sound	When inhaling, lift up both arms/hands with elbows fully extended from two sides to the shoulder level, move both arms/hands horizontally to the front and then towards the chest. Exhale as arms/hands move down slowly till the end of exhalation. Repeat the breath and body movement three times in a complete adjusting breath. Perform this movement before Movement 1, between each movement, and after Movement 6 (a total of seven times throughout the exercise).
Movement 1	Hsu [shh]	When inhaling, lift up both arms/hands the body to chest level, with the palms facing up and elbow joints fully extended, and then move the hands towards the chest. Exhale as the arms/hands move down. During the slow exhalation, chant “shh.” Repeat the sound and movement six times.
Movement 2	Her [her]	When inhaling, lift up both arms/hands near the body to the chest level with the palms facing up. Begin to exhale. During exhalation, chant “her” and continue to slowly move arms/hands up up to the eyebrow level. Inhale while moving arms/hands down. Convert to exhalation when the hands pass the chest level and continue to move arms/hands down. Repeat the sound and movement six times.
Movement 3	Hoo [who]	When inhaling, lift up both arms/hands near the body to chest level with palms facing up. Then, begin to exhale and chant “who” while slowly moving your left hand up and right hand down in a diagonal direction until the end of exhalation. Inhale and move left hand down and right hand up to the chest level again. Convert to exhalation and chant “who” while slowly moving the left hand up and the right hand down in a diagonal direction until the end of exhalation. Repeat the sound and movement three times.
Movement 4	Sss [sss]	When inhaling, lift up both arms/hands to the chest level with palms facing up. Begin to exhale. During exhalation, chant “sss” while slowly pushing the hands forward and then down to both sides until the end of exhalation. Repeat the sound and movement six times.
Movement 5	Chway [ch-way]	When inhaling, lift up both arms/hands through the back of trunk to the front of the chest as if holding a large ball. Begin to exhale. During exhalation, chant “chway” while slowly moving both hands down over an imaginary ball until touching the thighs. Bend both knees down slightly while you circle your hands down over the ball. Repeat the sound and movement six times.
Movement 6	See [see]	When inhaling, lift up both arms/hands near the body to the chest level with palms facing up. Begin to exhale. During exhalation, chant “see” and continue to slowly lift up hands above the head until the end of exhalation. Begin to inhale while slowly moving down arms/hands along the same path. Begin to exhale again when the hands pass the chest and continue to move arms/hands down until the end of exhalation. Repeat the sound and movement six times.
Throughout the entire exercise sequence, focus your mind on an important focal point, the so-called “Dan Tian” acupuncture point, which is located in the abdomen three finger widths below your belly button to establish and maintain the mind emptiness status.		

**Table 2:** Comparison of baseline characteristics of the experimental and control groups.

Characteristic	Experimental	Control
Age, y	61.8 ± 5.7	68.0 ± 5.3
Disease duration, y	8.0 ± 3.6	13.3 ± 3.6
Symptom duration, y	9.3 ± 4.2	13.9 ± 8.6
HY stage	2.7 ± 0.3	2.4 ± 0.5

Values are displayed as mean ± SD.

**Table 3:** Comparison of TNF-α levels in the experimental and the control groups pre- and post-intervention.

Assessment	TNF-α. pg/mL	
	Experimental	Control
Pre-intervention	13.8 ± 0.6	13.3 ± 3.3
Post-intervention	12.3 ± 1.6 *	15.1 ± 2.3

Values are displayed as mean ± SD. * *p* < 0.05.

**Table 4:** Comparison of Parkinson’s Disease Sleep Scale 2 (PDSS-2) scores and mean changes (Δ; Δ = post-score – baseline-score) of the experimental and control groups.

PDSS-2	Baseline		Mean Change (Δ)	
	Experimental	Control	Experimental	Control
Motor symptoms at night	4.5 ± 4.7	3.0 ± 2.5	−2.3 ± 4.6	2.8 ± 2.8
PD symptoms at night	6.0 ± 3.2	2.8 ± 2.2	−2.5 ± 3.8 *	2.3 ± 4.1
Disturbed sleep	8.3 ± 3.4	8.3 ± 5.3	−2.5 ± 4.1	4.0 ± 9.1
PDSS-2 total score	18.8 ± 8.1	14.0 ± 9.8	−7.3 ± 11.6 **	9.0 ± 12.8

Values are displayed as mean ± SD. * *p* < 0.05; ** *p* < 0.0005.
